# Factors Associated with Ever Being HIV-Tested in Zimbabwe: An Extended Analysis of the Zimbabwe Demographic and Health Survey (2010–2011)

**DOI:** 10.1371/journal.pone.0147828

**Published:** 2016-01-25

**Authors:** Kudakwashe Collin Takarinda, Lydia Kudakwashe Madyira, Mutsa Mhangara, Victor Makaza, Memory Maphosa-Mutsaka, Simbarashe Rusakaniko, Peter H. Kilmarx, Tsitsi Mutasa-Apollo, Getrude Ncube, Anthony David Harries

**Affiliations:** 1 AIDS and TB Department, Ministry of Health and Child Care, Harare, Zimbabwe; 2 National AIDS Council, Harare, Zimbabwe; 3 Department of Community Medicine, College of Health Sciences, University of Zimbabwe, Harare, Zimbabwe; 4 Centre for Research and Training in Clinical Epidemiology, College of Health Sciences, University of Zimbabwe, Harare, Zimbabwe; 5 Division of Global HIV/AIDS, Centers for Disease Control and Prevention (CDC), Harare, Zimbabwe; 6 International Union Against Tuberculosis and Lung Disease, Paris, France; 7 Department of Clinical Research, London School of Hygiene and Tropical Medicine, London, United Kingdom; Tulane University School of Public Health, UNITED STATES

## Abstract

**Introduction:**

Zimbabwe has a high human immunodeficiency virus (HIV) burden. It is therefore important to scale up HIV-testing and counseling (HTC) as a gateway to HIV prevention, treatment and care.

**Objective:**

To determine factors associated with being HIV-tested among adult men and women in Zimbabwe.

**Methods:**

Secondary analysis was done using data from 7,313 women and 6,584 men who completed interviewer-administered questionnaires and provided blood specimens for HIV testing during the Zimbabwe Demographic and Health Survey (ZDHS) 2010–11. Factors associated with ever being HIV-tested were determined using multivariate logistic regression.

**Results:**

HIV-testing was higher among women compared to men (61% versus 39%). HIV-infected respondents were more likely to be tested compared to those who were HIV-negative for both men [adjusted odds ratio (AOR) = 1.53; 95% confidence interval (CI) (1.27–1.84)] and women [AOR = 1.42; 95% CI (1.20–1.69)]. However, only 55% and 74% of these HIV-infected men and women respectively had ever been tested. Among women, visiting antenatal care (ANC) [AOR = 5.48, 95% CI (4.08–7.36)] was the most significant predictor of being tested whilst a novel finding for men was higher odds of testing among those reporting a sexually transmitted infection (STI) in the past 12 months [AOR = 1.86, 95%CI (1.26–2.74)]. Among men, the odds of ever being tested increased with age ≥20 years, particularly those 45–49 years [AOR = 4.21; 95% CI (2.74–6.48)] whilst for women testing was highest among those aged 25–29 years [AOR = 2.01; 95% CI (1.63–2.48)]. Other significant factors for both sexes were increasing education level, higher wealth status and currently/formerly being in union.

**Conclusions:**

There remains a high proportion of undiagnosed HIV-infected persons and hence there is a need for innovative strategies aimed at increasing HIV-testing, particularly for men and in lower-income and lower-educated populations. Promotion of STI services can be an important gateway for testing more men whilst ANC still remains an important option for HIV-testing among pregnant women.

## Introduction

Sub-Saharan Africa has the largest proportion of people living with the Human Immunodeficiency Virus (HIV) [1]. Data in 2013 indicated that 69% of HIV-infected people globally resided in this region although the region contributes only 12% of the global population [1]. Zimbabwe is one of the sub-Saharan countries worst affected by the HIV epidemic with an adult prevalence of 15% among adults aged 15–49 years [2].

Zimbabwe’s commitment to its HIV/AIDS response has resulted in a drive towards the elimination of AIDS and new HIV infections through various strategies. HIV testing and counseling (HTC) is the cross-cutting priority prevention strategy. Normative HIV testing in the population is viewed as a pivotal entry point into a number of other key strategies which include voluntary medical male circumcision (VMMC), prevention of mother-to-child transmission (PMTCT), HIV care and treatment, condom programming and behaviour change.

In Zimbabwe, HIV testing began in 1984 as part of routine screening of donated blood and blood products at the National Blood Transfusion Services of Zimbabwe (NBTSZ) and by 1990, diagnostic HIV testing had become available in clinical settings [3]. Whilst there are various models of HIV testing that are recommended by the World Health Organization (WHO) [1], the following models are more commonly available in Zimbabwe: i) stand-alone “opt-in” voluntary counselling and testing (VCT), ii) provider-initiated testing and counselling (PITC) in treatment settings i.e. TB, sexually transmitted infections (STI), in-patient or outpatient clinics, and iii) PITC in antenatal clinics. National HIV testing and counselling campaigns have also been adopted since 2010, which are aimed at improving the demand for and access to HIV-testing services at the community level [3]. In 2011, close to one percent (118,032 people) of the national population of 12,973,808 were tested for HIV as a result of a 10 day campaign of whom 49,911(42%) were males and 68,121(58%) were females [3].

The overall goal of Zimbabwe’s HTC strategy has been to ensure that 85% of all people starting at birth know their HIV status by 2015 [4]. However, this is under review as Zimbabwe considers moving towards a target of 90% in line with 90-90-90 targets set by the Joint United Nations Program for HIV/AIDS (UNAIDS) [5]. According to the Zimbabwe Demographic and Health Survey (ZDHS) 2010–11, only 36% and 57% of men and women respectively aged 15–49 years had ever been tested for HIV and received their results, regardless of them consenting to providing a blood specimen for HIV-testing during the current survey. This shows that a large percentage of the population is not aware of their HIV status despite the scale up in provision of HIV testing from 48% to 95% of all health facilities between 2007 and 2009 [6].

HIV testing, which results in knowledge of one’s HIV status, has been shown to result in reduced sexual risk behaviour [7][8][9][10] and HIV incidence,[11] particularly for those receiving community-based VCT.[8][11] In those who are HIV-infected, the dangers of not knowing one’s positive status include continued transmission of HIV in the community and a higher personal risk of developing opportunistic infections such as tuberculosis (TB) [12] and cryptococcal meningitis [13] which could lead to early death. Another important criterion for HIV testing is to enable HIV-positive people to access antiretroviral treatment (ART). Given the low uptake of HIV testing services in the country, there is need to describe the factors associated with persons ever having been HIV-tested. We therefore set out to determine i) socio-demographic factors and ii) sexual risk behaviors associated with ever being tested for HIV.

## Methods

### Study design

This study used secondary data from the 2010–11 ZDHS which was a descriptive cross-sectional survey with a stratified two-stage design.

### Ethics statement

Ethics approval for conducting the ZDHS was obtained from the Medical Research Council of Zimbabwe, the Institutional Review Board of ICF International and the Centers for Disease Control (CDC) whilst the secondary data analysis protocol was also reviewed and approved by The Union Ethics Advisory Group, International Union Against Tuberculosis and Lung Disease, Paris, France.

### Data and sources of data

The 2010–11 ZDHS is routinely conducted every 5 years with the aim of providing population and health indicator estimates at national and provincial levels. The data are available on http://www.dhsprogram.com/data/available-datasets.cfm and the dataset version used here is ZWIR62DT.ZIP. In the 2010–11 ZDHS, a nationally representative probability sample of 10,828 households was selected and interviewed using a stratified, two-stage cluster design. Details of the sampling procedures and how the survey was conducted are provided in more detail in the 2010–11 ZDHS report [2]. During the ZDHS, data were collected by trained interviewers using personal digital assistants (PDAs) through a gender specific questionnaire for men aged 15–54 years and women aged 15–49 years. Prior to data collection, all respondents aged ≥18 years provided written informed consent whilst those aged ≤18 years provided written informed parent consent as well as their own written informed assent. Technicians also collected finger prick blood spot specimens for laboratory testing of HIV for all men and women who voluntarily consented to the procedure as part of this anonymous serosurvey. It is important to note that only a subset of those who provided written informed consent to participate in the questionnaire also consented to having a finger prick blood spot specimen taken. The collection procedure of the specimens, the consent process, transportation of the specimens and their laboratory analysis are outlined in the ZDHS 2010–11 report [2]. Respondents were not informed of their HIV status and their results were kept confidential and used only for survey purposes.

### Study population

Our analysis was limited to women aged 15–49 years and men aged 15–54 years who voluntarily participated in the questionnaire administration and consented to a blood specimen draw as part of the anonymous serosurvey in the 2010–11 ZDHS which was conducted over 6 months from September 2010 until March 2011.

### Definition of Terms

In this study the outcome variable, ‘ever been HIV-tested’ was defined as having accessed HIV-testing services and received their results at least once in their lifetime prior to this survey. The wealth index, used in the survey and adopted from Filmer and Pritchett [14], was based on information collected from respondents on several items that measure household ownership of consumer durables which tend to be correlated with household wealth status, and these were used to quantify differences in household economic status. This wealth index variable was a continuous variable which was divided into 5 equal quintiles which were ranked from the lowest category (termed *‘poorest quintile’*) to the highest category (termed *richest quintile’)*. *‘Current union’* status referred to those who were married or cohabiting whilst *‘formerly in union’* referred to those who were divorced, widowed or separated. A self-reported history of sexually transmitted infections (STIs) was obtained from both men and women. For a birth in the past 5 years, women were asked about attendance at antenatal care (ANC) whilst in order to limit recall bias, men were asked if their partner had attended ANC for births in the past 2 years.

Religion was categorized into Christians, Apostolic sects and ‘other minority religions/none’ which referred to the African traditional religious, Muslims, other minority religions and those who are not religious. Comprehensive knowledge of HIV was also assessed, and this was defined as: i) knowing that correct and consistent use of condoms during sexual intercourse and having just one uninfected faithful partner can reduce the chances of getting HIV, ii) knowing that a healthy-looking person can have HIV, and iii) rejecting the 2 most common local misconceptions about HIV transmission which are that HIV can be transmitted by mosquito bites and that HIV can be transmitted by supernatural means. Finally, in this study, comprehensive accepting attitudes towards HIV meant i) willingness to care for a family member with AIDS in the respondent’s home, ii) willingness to buy fresh vegetables from a shopkeeper who has HIV, iii) accepting that a female teacher who has HIV but is not sick should be allowed to continue teaching and iv) not keeping any secrets that a family member was infected with HIV.

### Statistical analysis

Statistical analyses were performed using Stata/IC 13.0 (StataCorp, 2013, Stata Statistical Software: Release 13.0, College Station, TX: StataCorp LP). All data presented were weighted using HIV weights in order to adjust for the sample design and differences in response rates to the ZDHS interview and the HIV testing component [2]. Separate logistic regression models for men and women were used to calculate univariate and multivariate-adjusted odds ratios and their 95% confidence intervals (CI) whilst adjusting for potential confounding. First, all variables which had a p-value ≤ 0.25 in the univariate analysis were selected into model 1 of the multivariate logistic model. After which, a subsequent multivariate logistic model was restricted to those variables that remained significant in the first multivariate logistic model with p-values ≤ 0.25. Multicollinearity was checked for, in the multivariate logistic regression models, using the *collin* command in Stata whereby variables which had a variance inflation factor (VIF) of ≥10 or tolerance (defined as 1/VIF) < 0.1 where excluded from the model, in specific “age at first sex.” Interaction was also assessed between “education and wealth” and “urban/rural residence and province” using the *testparm* command in Stata, of which rural/urban residence was excluded from the final model because of interaction with province.

Re-categorization of some variables was also done to avoid collinearity until the model of best fit was achieved. Separate multivariate models for both men and women were also generated to obtain adjusted odds ratios for the variables “*consistent condom use in the past 12 months*” and “*number of lifetime sexual partners*” since these variables are made up of a subset of sexually active individuals.

## Results

Information was collected from 9,756 households, from which 9,831 women aged 15–49 years and 8,723 men aged 15–54 years were eligible for interview and blood sample collection. Of these, interviews were completed for 9,143 (93%) of women and 7,502 (86%) of men. Our analysis is limited to the 7,313 (80%) women and 6,584 (88%) men who also provided blood samples for the anonymous serosurvey following questionnaire completion. All further data presented are weighted to adjust for the sample design and differences in response rates to the ZDHS interview and the HIV testing component. **Table 1** shows the percent distribution of socio-demographic and sexual risk behaviours. Overall, more women had ever been tested for HIV compared with men among those who were eligible for this analysis (61% versus 39%; p<0.001).

**Table 1:** Weighted numbers and percent distribution of socio-demographic and sexual risk characteristics among male and female respondents, ZDHS 2010–11.

Background characteristics	Women		Men	
	N	%	N	%
*Age*				
15–19	1,553	21.2	1,569	23.8
20–24	1,463	20.0	1.204	18.3
25–29	1,354	18.5	1.082	16.4
30–34	1,010	13.8	844	12.8
35–39	843	11.5	712	10.8
40–44	588	8.1	506	7.7
45–49	501	6.9	333	5.1
50–54	-	-	334	5.1
*Education level*				
no education	168	2.3	67	1.0
Primary	2,156	29.5	1,603	24.4
Secondary	4,688	64.1	4,495	68.3
Higher	300	4.1	419	6.4
*Wealth index*				
Poorest	1,375	18.8	1,092	16.6
Poorer	1,411	19.3	1,259	19.1
Middle	1,457	19.9	1,351	20.5
Richer	1,527	20.9	1,465	22.3
Richest	1,544	21.1	1,419	21.5
*Residence*				
Urban	2,297	31.4	1,966	29.9
Rural	5,015	68.6	4,618	70.1
*Province*				
Manicaland	1,005	13.7	927	14.1
Mashonaland Central	768	10.5	746	11.3
Mashonaland East	740	10.1	690	10.5
Mashonaland West	863	11.8	865	13.1
Matabeleland North	353	4.8	322	4.9
Matabeleland South	407	5.6	346	5.3
Midlands	939	12.8	860	13.1
Masvingo	757	10.4	566	8.6
Harare	1,122	15.3	967	14.7
Bulawayo	360	4.9	295	4.5
*Religion*				
Christians	3,917	54.0	2,971	45.1
Apostolic sect	2,843	39.0	1,800	27.3
Traditional	49	0.7	316	4.8
None	466	6.4	1,455	22.1
Muslim	36	0.5	37	0.6
Other	2	0.03	5	0.1
*Union status*				
never in union	1,694	23.2	2,853	43.3
currently in union	4,569	62.5	3,428	52.1
formerly in union	1,049	14.0	303	4.6
*ANC visit*				
no birth in last 5yrs	3,700	50.6	-	-
no visit	290	4.0	-	-
yes: visited	3,323	45.4	-	-
***Partner visited ANC for birth in past 2 years***				
no birth	-	-	3,126	47.5
birth, but in >2yrs	-	-	1,743	26.5
no ANC visit/don't know	-	-	208	3.2
had ANC visit	-	-	1,507	22.9
*Age at first sex*				
never had sex	1,269	17.4	1,573	23.9
<16 yrs	976	13.4	521	7.9
16–17yrs	1,737	23.8	890	13.5
18–19	1,615	22.1	1,245	18.9
20+	1,652	22.6	2,238	34.0
don't know	63	0.9	117	1.8
*Ever had paid sex*				
No	-	-	5,417	82.3
Yes	-	-	1,167	17.7
*No*. *of lifetime sexual partners*				
0	1,269	17.4	1,573	23.9
1	3,867	52.9	882	13.4
2	1,345	18.4	867	13.2
3–4	608	8.3	1,337	20.3
5	223	3.1	1,925	29.2
*Consistent condom use in the past last 12 months*				
Never had sex	2,327	31.8	2,078	31.6
No	482	6.6	3,656	55.5
Yes	4,504	61.6	850	12.9
Had an STI in the past 12 months				
No	7,083	96.9	6,411	97.4
Yes	224	3.1	166	2.5
don't know	6	0.1	7	0.1
*HIV status*				
HIV-negative	6,018	82.2	5,750	87.3
HIV-positive	1,295	17.7	834	12.7
*Comprehensive knowledge of HIV*				
No	3,204	43.8	3,075	46.7
Yes	4,109	56.2	3,509	53.3
*Comprehensive accepting attitudes towards HIV*				
No	4,501	61.6	4,057	61.6
Yes	2,812	38.5	2,528	38.4
**Total**	**7,313**	**100**	**6,584**	**100**

ANC = ante-natal care; HIV = human immunodeficiency virus; ZDHS = Zimbabwe Demographic Health Survey; STI = sexually transmitted infections

NB: Weighted numbers and percentages in the table above may not always add up to the totals shown in the last row of the table because of rounding-off errors.

### Factors associated with uptake of HIV testing

**Tables 2** and **3** show socio-demographic and sexual risk factors associated with ever being HIV tested among women and men respectively. Women aged 20–24 years had the highest odds of ever being tested [aOR = 2.01; 95% CI (1.63–2.48)] compared to those aged 15–19 years whilst an increasing odds of declining testing was noted from ages 25 to 39 years. Also among men, being 20 years and older was significantly associated with a higher odds of ever being HIV tested compared to those aged 15–19 years, with men aged 45–49 years being particularly more likely to have ever been tested [aOR = 4.21; 95% CI (2.74–6.48)]. The odds of ever having been HIV-tested also increased significantly with higher education level in both sexes: compared to those with primary or no education, men and women with higher education levels were more likely to have ever received testing i.e. [aOR = 2.26; 95% CI (1.97–3.61)] and [aOR = 2.21; 95% CI (1.54–3.16)] respectively. Likewise the odds of ever having been tested increased with increasing wealth index compared to the lowest wealth index for both men and women; notably in the richest wealth quintile for men [aOR = 1.91; 95% CI (1.5–2.44)] and the richest wealth quintile for women [aOR = 1.49; 95% CI (1.14–1.95)].

**Table 2:** Sociodemographic and sexual risk factors associated with ever being HIV-tested among female respondents, ZDHS 2010–11.

Background characteristics	N	%	OR (95% CI); p-value	AOR (95% CI); p-value
*Age*				
15–19	1,553	28.2	reference	reference
20–24	1,463	70.4	6.04 (5.05–7.23); <0.001	1.82 (1.47–2.24); <0.001
25–29	1,354	78.9	9.49 (7.88–11.44); <0.001	2.01 (1.63–2.48); <0.001
30–34	1,010	73.1	6.90 (5.72–8.33); <0.001	1.53 (1.22–1.92); <0.001
35–39	843	67.7	5.33 (4.39–6.48); <0.001	1.37 (1.07–1.74); 0.011
40–44	589	60.8	3.95 (3.17–4.91); <0.001	1.25 (0.97–1.61); 0.084
45–49	501	50.6	2.61 (2.08–3.27); <0.001	1.23 (0.93–1.62); 0.141
50–54	-	-	-	-
*Education level*‡				
primary or less	2,324	55.8	reference	reference
Secondary	4,688	62.8	1.34 (1.19–1.51); <0.001	1.69 (1.45–1.97); <0.001
Higher	301	70.9	1.93 (1.45–2.56); <0.001	2.21 (1.54–3.16); <0.001
*Wealth index*				
Poorest	1,375	57.5	reference	reference
Poorer	1,411	59.0	1.06 (0.9–1.26); 0.475	1.13 (0.94–1.36); 0.198
Middle	1,456	63.2	1.26 (1.05–1.52); 0.013	1.46 (1.17–1.84); 0.001
Richer	1,527	65.3	1.39 (1.15–1.68); 0.001	1.54 (1.19–1.99); 0.001
Richest	1,544	59.3	1.07 (0.89–1.29); 0.449	1.49 (1.14–1.95); 0.003
*Residence*				
Urban	2,297	62.1	reference	reference
Rural	5,016	60.4	0.93 (0.82–1.06); 0.292	-
*Province*				
Manicaland	1,005	62.6	reference	reference
Mashonaland Central	767	62.9	1.01 (0.74–1.39); 0.928	1.00 (0.68–1.46); 0.987
Mashonaland East	740	60.1	0.90 (0.66–1.23); 0.512	0.77 (0.55–1.09); 0.146
Mashonaland West	863	61.7	0.96 (0.68–1.37); 0.832	0.90 (0.60–1.36); 0.618
Matabeleland North	353	63.3	1.03 (0.74–1.44); 0.862	1.31 (0.86–2.01); 0.210
Matabeleland South	407	64.2	1.07 (0.82–1.40); 0.622	1.29 (0.91–1.82); 0.152
Midlands	939	53.6	0.69 (0.51–0.93); 0.016	0.60 (0.43–0.86); 0.005
Masvingo	757	60.1	0.90 (0.67–1.21); 0.492	0.86 (0.60–1.22); 0.387
Harare	1,122	62	0.98 (0.74–1.29); 0.872	0.95 (0.69–1.30); 0.733
Bulawayo	360	63.3	1.03 (0.76–1.40); 0.837	1.09 (0.76–1.56); 0.635
*Religion*				
Christians	3,917	60.2	reference	Reference
Apostolic sect	2,843	62.2	1.09 (0.97–1.22); 0.169	0.98 (0.84–1.14); 0.783
Other religions/none*	553	59.5	0.97 (0.80–1.18); 0.746	0.74 (0.60–0.91); 0.005
*Union status*				
never in union	1,695	27.8	reference	Reference
currently in union	4,569	70.8	6.30 (5.46–7.28); <0.001	1.64 (1.27–2.13); <0.001
formerly in union	1,049	71.5	6.53 (5.43–7.86); <0.001	2.03 (1.52–2.72); <0.001
*ANC visit*				
no visit	290	46.7	reference	Reference
yes: visited	3,323	82.1	5.23 (3.93–6.95); <0.001	5.48 (4.08–7.36); <0.001
no birth in last 5yrs	3,700	43.0	0.86 (0.66–1.12); 0.262	1.44 (1.10–1.89); 0.009
*Age at first sex*				
never had sex	1,269	16.1	reference	Reference
<16 yrs	977	64.4	9.46 (7.57–11.84); <0.001	-
16–17yrs	1,737	68.8	11.5 (9.38–14.11); <0.001	-
18–19	1,615	73.3	14.36 (11.84–17.41); <0.001	-
20+	1,652	73.3	14.35 (11.65–17.68); <0.001	-
don't know	63	52.5	5.78 (3.45–9.68); <0.001	-
*No*. *of lifetime sexual partners among those that ever had sex*				
1	3,867	69.1	reference	reference
2	1,345	70.7	1.08 (0.93–1.25); <0.311	1.04 (0.89–1.22); 0.629
3–4	609	75.1	1.35 (1.09–1.62); 0.006	1.35 (1.06–1.73); 0.017
≥5	223	77.4	1.53 (1.09–2.17); 0.016	1.48 (1.03–2.15); 0.036
*Consistent condom use in the past last 12 months among those that ever had sex*	* *			
No	482	79	reference	reference
Yes	4504	70.2	0.63 (0.49–0.80); <0.001	0.55 (0.41–0.73); <0.001
Had an STI in the past 12 months†				
No	7089	60.4	reference	reference
Yes	224	77.9	2.31 (1.64–3.27); <0.001	1.37 (0.96–1.94); 0.081
*HIV status*				
HIV-negative	6,018	58.2	reference	reference
HIV-positive	1,295	73.6	2.00 (1.75–2.30); <0.001	1.42 (1.20–1.69); <0.001
*Comprehensive knowledge of HIV*				
No	3,204	53.6	reference	reference
Yes	4,109	66.6	1.73 (1.56–1.92); <0.001	1.44 (1.28–1.63); <0.001
*Comprehensive accepting attitudes towards HIV*				
No	4,501	57.8	reference	reference
Yes	2,812	65.9	1.41 (1.25–1.59); <0.001	1.24 (1.08–1.42); 0.002
**Total**	**7,313**	**60.9**	** **	** **

ANC = ante-natal care; HIV = human immunodeficiency virus; ZDHS = Zimbabwe Demographic Health Survey; STI = sexually transmitted infections

‡ Only 2.4% of all women have no education hence "no education" and "primary education " have been combined

* "other religion" refers to traditional religion, Muslims and other minority religions

† <1% of those classified as "not having an STI in the last 6 months" reported being unsure of having an STI in the last 12 months

NB: Separate multivariate models have been generated to obtain adjusted odds ratios for the variables “consistent condom use in the past 12 months” and “number of lifetime sexual partners” since these variables are made up of a subset of sexually active individuals.

**Table 3:** Sociodemographic and sexual risk factors associated with ever being HIV-tested among male respondents, ZDHS 2010–11.

Background characteristics	N	%	OR (95% CI); p-value	AOR (95% CI); p-value
*Age*				
15–19	1,569	11.9	reference	reference
20–24	1,204	36.0	4.15 (3.30–5.23); <0.001	2.50 (1.94–3.23); <0.001
25–29	1,082	50.7	7.60 (6.01–9.62); <0.001	3.29 (2.43–4.45); <0.001
30–34	844	52.3	8.08 (6.37–10.24); <0.001	3.17 (2.24–4.48); <0.001
35–39	712	50.1	7.42 (5.82–9.47); <0.001	2.81 (1.97–4.02); <0.001
40–44	506	52.5	8.16 (6.23–10.69); <0.001	2.82 (1.95–4.07); <0.001
45–49	333	57.7	10.08 (7.22–14.08); <0.001	4.21 (2.74–6.48); <0.001
50–54	334	44.0	5.80 (4.29–7.85); <0.001	2.62 (1.73–3.98); <0.001
*Education level*‡				
primary or less	1,670	29.9	reference	reference
Secondary	4,495	39.9	1.56 (1.35–1.8); <0.001	1.57 (1.34–1.84); <0.001
Higher	419	67.2	4.8 (3.64–6.33); <0.001	2.66 (1.97–3.61); <0.001
*Wealth index*				
Poorest	1,092	33.4	reference	reference
Poorer	1,258	31.1	0.90 (0.73–1.11); 0.328	0.97 (0.77–1.22); 0.801
Middle	1,350	36.6	1.15 (0.93–1.42); 0.186	1.26 (1.01–1.57); 0.037
Richer	1,465	41.8	1.43 (1.15–1.78); 0.001	1.43 (1.12–1.83); 0.004
Richest	1,419	50.1	2.00 (1.63–2.46); <0.001	1.91 (1.5–2.44); <0.001
*Residence*				
Urban	1,966	44.2	reference	reference
Rural	4,618	36.9	0.74 (0.64–0.86); <0.001	-
*Province*				
Manicaland	927	43.9	referemce	Referemce
Mashonaland Central	746	41.7	0.91 (0.60–1.4); 0.677	1.12 (0.77–1.64); 0.560
Mashonaland East	690	36.0	0.72 (0.47–1.10); 0.131	0.80 (0.53–1.20); 0.283
Mashonaland West	865	42.1	0.93 (0.62–1.38); 0.707	1.01 (0.7–1.47); 0.938
Matabeleland North	322	39.7	0.84 (0.51–1.38); 0.492	1.27 (0.80–2.02); 0.315
Matabeleland South	346	28.2	0.50 (0.32–0.80); 0.004	0.67 (0.45–0.99); 0.043
Midlands	860	30.6	0.56 (0.38–0.83); 0.003	0.53 (0.38–0.75); <0.001
Masvingo	566	36.1	0.72 (0.48–1.09); 0.119	0.76 (0.52–1.10); 0.149
Harare	967	42.1	0.93 (0.63–1.36); 0.699	0.63 (0.45–0.89); 0.010
Bulawayo	295	47.7	1.16 (0.76–1.77); 0.477	0.78 (0.52–1.17); 0.235
*Religion*				
Christians	2,971	42.5	Reference	Reference
Apostolic sect	1,800	35.9	0.76 (0.66–0.87); <0.001	0.86 (0.72–1.02); 0.081
Other religions/none*	1,813	36.5	0.78 (0.67–0.90); 0.001	0.64 (0.54–0.76); <0.001
*Union status*				
never in union	2,853	22.8	Reference	Reference
currently in union	3,428	51.7	3.62 (3.16–4.14); <0.001	1.65 (1.17–2.34); 0.004
formerly in union	303	49.9	3.38 (2.57–4.43); <0.001	1.52 (1.03–2.24); 0.036
*Partner visited ANC for birth in past 2 years* ǂ				
no birth	3,126	25.1	reference	Reference
had ANC visit	1,507	54.3	3.55 (3.05–4.13); <0.001	1.31 (1.00–1.72); 0.048
no ANC visit/don't know	208	35.5	1.65 (1.18–2.29); 0.003	0.65 (0.45–0.94); 0.023
birth, but in >2yrs	1,743	51.4	3.15 (2.74–3.62); <0.001	1.08 (0.83–1.42); 0.561
*Age at first sex*				
never had sex	1,573	14.8	reference	Reference
<16 yrs	521	37.5	3.45 (2.65–4.50); <0.001	-
16–17yrs	890	41.3	4.05 (3.24–5.07); <0.001	-
18–19	1,245	45.6	4.82 (3.93–5.90); <0.001	-
20+	2,238	51.7	6.17 (5.12–7.43); <0.001	-
don't know	117	43.9	4.51 (2.80–7.27); <0.001	-
*Ever had paid sex*				
No	5,417	36.9	reference	Reference
Yes	1,167	49.1	1.65 (1.43–1.90); <0.001	0.94 (0.79–1.11); 0.441
*No*. *of lifetime sexual partners among those that have had sex*				
1	882	39.8	reference	reference
2	867	49.9	1.50 (1.21–1.86); <0.001	1.29 (1.03–1.62); 0.027
3–4	1,337	43.6	1.17 (0.97–1.41); 0.106	0.90 (0.73–1.10); 0.292
5	1,925	50.5	1.54 (1.28–1.87); <0.001	1.10 (0.88–1.37); 0.386
*Consistent condom use in the past last 12 months among those that ever had sex*				
No	3,656	48.6	reference	reference
Yes	850	47.0	0.94 (0.79–1.11); 0.445	1.42 (1.14–1.78); 0.002
Had an STI in the past 12 months†				
No	6,418	38.5	reference	Reference
Yes	166	59.1	2.30 (1.55–3.42); <0.001	1.86 (1.26–2.74); 0.002
*HIV status*				
HIV-negative	5,750	36.7	reference	Reference
HIV-positive	834	55.2	2.13 (1.81–2.50); <0.001	1.53 (1.27–1.84); <0.001
*Comprehensive knowledge of HIV*				
No	3,075	33.4	reference	Reference
Yes	3,509	44.0	1.56 (1.37–1.79); <0.001	1.09 (0.94–1.26); 0.268
*Comprehensive accepting attitudes towards HIV*				
No	4,056	35.7	reference	Reference
Yes	2,528	44.5	1.44 (1.27–1.64); <0.001	1.12 (0.97–1.29); 0.12
**Total**	**6584**	**39.1**	** **	

ANC = ante-natal care; HIV = human immunodeficiency virus; ZDHS = Zimbabwe Demographic Health Survey; STI = sexually transmitted infections

‡ Only 1.3% 2of all men have no education hence "no education" and "primary education” have been combined

* "other religion" refers to traditional religion, Muslims and other minority religions

ǂ Men who are not in union were asked if they had a pregnant partner, and so are assumed to have a pregnant partner

† 1.1% of those classified as "not having an STI in the last 6 months" reported being unsure of having an STI in the last 12 months

NB: Separate multivariate models have been generated to obtain adjusted odds ratios for the variables “consistent condom use in the past 12 months” and “number of lifetime sexual partners” since these variables are made up of a subset of sexually active individuals.

In comparison to Manicaland province, the odds of ever being tested were similar across all provinces excluding the Midlands province where the odds of ever having been tested were lower in both women [aOR = 0.60; 95% CI (0.43–0.86)] and men [aOR = 0.53; 95% CI (0.38–0.75)] and also lower in Harare province [aOR = 0.63; 95% CI (0.45–0.89)] among men. A lower odds of having been tested was also noted in both men [aOR = 0.64; 95% CI (0.54–0.76)] and women [aOR = 0.74; 95% CI (0.60–0.91)] of minority religions or those who were non-religious in comparison to those of Christian faith. Eight percent of all female respondents and 28% of all male respondents were of a minority religion or were non-religious.

Those women [aOR = 1.64; 95% CI (1.27–2.13)] and men [aOR = 1.65; 95% CI (1.17–2.34)] who were currently in union or women [aOR = 2.03; 95% CI (1.52–2.72)] and men [aOR = 1.52; 95% CI (1.03–2.24)] formerly in union were more likely to have ever been tested in comparison to those who were never in union. Women who had attended ANC in the past 5 years had a higher odds of ever being HIV-tested [aOR = 5.48; 95% CI (4.08–7.36)] compared to those who never visited ANC, and similarly men who reported their partner having attended ANC in the past 2 years also had a borderline higher odds of ever being tested [aOR = 1.31; 95% CI (1.00–1.72)] when compared to those men with spouses who had not given birth in the past 2 years.

The odds of ever having been HIV-tested increased with increasing numbers of lifetime sexual partners among women and were highest among those reporting ≥5 lifetime sexual partners [aOR = 1.48; 95% CI (1.03–2.15)] whilst amongst men, only those who had two lifetime sexual partners had the highest odds of ever having been tested [aOR = 1.29; 95% CI (1.03–1.62)] in comparison to those who reported having had one lifetime sexual partner. Reporting an STI history in the past 12 months was only associated with ever being tested among men [aOR = 1.86; 95% CI (1.26–2.74)].

Whilst the odds of ever having been tested was higher among men reporting consistent condom use in the past 12 months [aOR = 1.42; 95% CI (1.14–1.78)], among women those reporting consistent use of condoms in the past 12 months were less likely to have ever been tested [aOR = 0.55; 95% CI (0.41–0.73)] compared to those who reported inconsistent condom use in the past 12 months. Though the odds of ever being tested was higher among those diagnosed as HIV-infected during the anonymous serosurvey for both women [aOR = 1.42; 95% CI (1.20–1.69)] and men [aOR = 1.53; 95% CI (1.27–1.84)] compared to those who were HIV-negative, only 74% of the women and 55% of men who were diagnosed as HIV-infected had ever been tested. Overall, HIV sero-prevalence was 17.7% among women and 12.7% among men. Lastly, having comprehensive knowledge of HIV [aOR = 1.44; 95% CI (1.28–1.63)] and comprehensive accepting attitudes towards HIV [aOR = 1.24; 95% CI (1.08–1.42)] were associated with ever having been tested among women although there was no association among men.

## Discussion

This study provides information on HIV testing from a nationally representative sample of adult men and women in Zimbabwe. Our study findings show that fewer men than women had ever been tested for HIV, and similar trends were noted in a review of 23 out of 29 demographic and health surveys conducted in other sub-Saharan African countries by Staveteig et al [15] although Zimbabwe had the highest difference between the two sexes next to Lesotho. Given the high prevalence of HIV in Zimbabwe, such low proportions of men and women reporting ever having been tested for HIV remains a barrier to early HIV treatment and care among those HIV-infected and this could impact negatively on their survival and result in poor ART response upon initiation [16][17]. Studies from both resource-limited settings [18][19][20] and well-resourced settings [16][21][22] have shown that men tend to have more advanced HIV disease upon initiation of ART, which is closely associated with late diagnosis of HIV infection. A recent review of ART programme data in Zimbabwe also revealed that men initiate ART later than women and that male gender is associated with patient attrition [23]. Whilst HIV testing is low among men, Zimbabwe fairs better in terms of the proportion of women who have ever been tested for HIV when compared to other regional countries such as Mozambique, Zambia, Namibia and Swaziland which reported 33%, 35%, 36% and 51% respectively in the most recent demographic and health surveys conducted in these countries [15].

We noted in our results that proportions of women ever tested for HIV-were highest among women in the 20–29 year age group whilst steadily declining with increasing age from 30 years old. This trend closely correlates with age-specific fertility rates reported in the 2010–2011 ZDHS [2] where childbearing peaks at 212 and 194 live-births per 1,000 women for those aged 20–24 and 25–29 years before dropping with older age. This further strengthens our impression that women in the 20–29 year age group may have accessed HIV testing during ANC visits. Furthermore it is important to take note of the high uptake of HIV testing, 70% and 79% of women aged 20–24 and 25–29 years respectively, which are the most numerous child-bearing age groups in Zimbabwe. On the other hand, proportions of men who were ever tested were higher among those aged ≥20 years who are sexually active and hence may have a higher HIV-risk perception given that the median age of sexual debut among Zimbabwean men is 20.6 years [2]. Compared to men in Zimbabwe, women have an earlier age of sexual debut at 18.9 years [2] and hence are at an earlier risk of HIV-infection although our data tends to indicate the odds of HIV-testing are highest among those attending ANC which may mean they are infected much earlier but learn of their status once pregnant. This possibility is strengthened by the fact that in 2013 almost 60% of all new HIV infections globally among young people aged 15–24 years occurred among adolescent girls and young women and that 80% of these 15–24 year old women reside in sub-Saharan Africa.[24] Challenges, however, exist concerning access to HIV testing for persons <18 years old as they require parental consent which may explain the low testing uptake in those aged 15–19 years for both sexes.

From our study we found that antenatal care visits were a critical gateway to HIV testing and counseling among women. Demographic health surveys conducted in other resource-limited countries have also shown equally high proportions of pregnant women tested for HIV in ANC [15]. This is most likely attributed to integration of ‘opt-out’ provider-initiated testing and counseling in ANC as an entry point into prevention-of-mother-to-child HIV transmission services and HIV treatment and care for HIV-infected pregnant mothers. In Zimbabwe, the routine offer of HIV testing in antenatal sites was first piloted in 2005 and findings showed high rates of HIV testing and acceptability in both rural [25] and urban settings [26]. In Sub-Saharan Africa, the scale-up of integrated HIV screening within routine health care settings in resource-limited countries has also been credited for the remarkable scale-up of the proportion of women who have been tested in ANC between the last two successive demographic health surveys, particularly in Malawi, Rwanda, Senegal, Tanzania, Uganda and Zimbabwe [15].

There was a borderline higher odds of ever being tested for HIV amongst men whose partners had attended ANC for a birth in the two years prior to the survey compared to those whose partner did not give birth or where there was no partner. Whilst this association may be due to chance, and is therefore interpreted with caution, it may be the case that increased testing in this group is linked to increasing advocacy in Zimbabwe directed at the male partner to attend ANC visits with their spouse. Male partner attendance of ANC has been shown elsewhere to be an acceptable strategy for increasing male involvement in PMTCT, hence boosting male HIV testing [27] and promoting HIV prevention interventions [28]. In a Ugandan study among men, spousal communication about HIV prevention was associated with self-reported HIV testing whilst a greater level of interest in learning how to help one's partner have a safe pregnancy increased the likelihood of willingness to test for HIV [29]. Despite the potential of increased HIV testing among males who accompany their pregnant spouses to ANC, much more remains to be achieved as our results show that only 55% of men with partners who visited ANC in the past 2 years reported being HIV-tested. In one Zimbabwean study, [26] encouragement of women receiving HIV testing in ANC to bring their male partners, showed that only 7% of men attended ANC and likewise in a Malawi study [30], 8% of HIV-infected partners undertook HIV testing. This low proportion of HIV testing among male partners may be linked to ANC settings being female centred environments and therefore alternative arrangements such as provision of places for men to congregate while their partners are giving birth or extending opening hours for clinics to allow men to attend after work may be innovative strategies to lure more men into getting tested.

Similar to other resource-limited settings, [31][32][33] HIV testing increased with education level and wealth status in both men and women. Further reference to data on women in the 2010–11 ZDHS report [2] shows that for declining educational level and declining wealth status, the total fertility rate increases, and yet there is a decreasing trend in the proportion of women who received ANC care from a skilled provider for the last live birth in the 5 years preceding the survey. Furthermore, a sub-analysis in the 2010–11 ZDHS on content of ANC services among women who reported attending ANC also shows a decreasing trend in the proportion of women who had a blood sample taken with decreasing education and decreasing wealth status. Closely related to this are decreasing trends in the proportions a) delivered in a health facility; b) delivered by a skilled health worker; and, c) with a postnatal check-up in the first 2 days after birth with decreasing educational level and decreasing wealth index, [2] which are all opportunities for receiving an HIV test. This indicates missed opportunities for HIV testing among those with lower education levels and lower wealth status.

Lower proportions of HIV testing were noted for both men and women in the Midlands province and in Harare province for men. Reasons for this when compared with all other provinces are unclear and need further exploration. A plausible reason for why HIV testing was lower among men and women of minority religions or those who were non-religious is also unclear. It is, however, important to note that these minority religion or non-religious groups constituted a small proportion of quite heterogeneous groups; hence it is difficult to explain this trend. Future qualitative studies among these groups may help to explain this low proportion of HIV testing. In general, barriers to accessing healthcare services in relation to religion have been noted among people of the Apostolic Sects whose religious beliefs shun seeking medical care and education of women and often result in early marriage [34][35][36] and higher risk of maternal mortality.[37] However, one qualitative study in Zimbabwe showed that members of these Apostolic groups secretly accessed modern healthcare services in violation of their church doctrine or they sought out special outreach initiatives that enabled them to obtain medical assistance incognito [38]. This may explain why their access to HIV testing services does not differ in comparison to people of other religious beliefs for both sexes.

The proportions of women tested for HIV were higher among those who had increasing lifetime sexual partners. Among men, a higher odds of testing was only noted among those who had two lifetime sexual partners in comparison with one lifetime sexual partner whilst there were no differences between men who reported ever paying for sexual intercourse and those who never paid for sex. Though men who reported consistently using condoms in the past 12 months were more likely to have ever been HIV tested, on the contrary, women reporting consistent condom use in past 12 months were less likely to have ever been tested. Other than biases that may arise from misreporting of information on sexual behaviours, cross-sectional studies prohibit the distinction between cause and effect [39] and as such it is difficult to establish whether the reported sexual risk behaviours preceded HIV testing or vice-versa. In one local prospective study, women who tested HIV-positive reported increased condom use in their regular relationships whilst those who tested HIV-negative were more likely to adopt risky behaviours in terms of numbers of previous or concurrent partnerships [40]. In another meta-analysis of 11 independent studies in the United States, [41] the prevalence of high-risk sexual behaviours was reduced substantially after people became aware that they were HIV-infected. In view of this, it appears that men with high risk sexual behaviour, particularly those reporting a history of paid sex and three or more lifetime sexual partners, are not accessing HIV-testing services more frequently than the low risk groups and this needs improvement. This former group has a higher exposure to HIV given that female sex workers have a reported HIV prevalence as high as 56%,[42] which is three times higher than the national adult prevalence.

Men who reported having an STI in the past 12 months were more likely to have been tested and this may present opportunities for scaling up testing among men through promotion of STI services given that STIs are closely associated with acquiring HIV. The odds of ever being tested were higher in those found to be HIV-infected in this anonymous serosurvey for both sexes. Despite this, just over a quarter of women and about half of men who were HIV-infected had never been tested. This highlights missed opportunities for diagnosis of HIV-infected individuals and their subsequent entry into HIV treatment and care services. On the other hand, whilst a significant proportion of HIV-infected individuals were previously tested it is unclear whether HIV infection preceded testing or vice-versa. For instance, one South African survey [43] revealed an increasing burden of undiagnosed HIV with an increasing time period since the last HIV test among those individuals that had been previously tested. This underscores the need for frequent retesting so that individuals know their true HIV status. It may therefore be beneficial if future demographic health surveys also ask respondents who were previously tested what their current HIV status is with the aim to identify the proportion that are still unaware of their true HIV status.

A key limitation of this study is recall bias and social desirability bias. Reporting of STIs, number of lifetime sexual partners and HIV-testing were all self reported and could not be validated in another manner. Whilst it is possible there are other important reasons for people not getting tested such as stock-outs of HIV test kits at health clinics, our study did not address this area. The interpretation of our results was also limited due to the cross-sectional design of the study. However, the survey design and sampling enables us to draw conclusions which are nationally representative and can be inferred to the general Zimbabwean population.

## Conclusions

Antenatal care visits and other maternal health services are an important means of accessing HIV testing services for women and also for men with pregnant partners through improved male involvement. Given the increased proportions ever tested among those with higher education levels and increased wealth status, policymakers should look into strategies targeted at scaling up HIV testing in the lower level-income and lower-educated populace. In general, HIV testing was lower among men and younger people, and this calls for policymakers to devise or strengthen strategies aimed at improving HIV testing among these groups. For younger people, this includes re-examining the age of consent for HIV testing and counseling.

Feasible options targeted at men include continued integration of HIV testing in provision of STI services and incorporation of HIV-testing as an entry-point to male circumcision in public health facilities. Since Zimbabwe introduced voluntary medical male circumcision in 2009, there have been 155,660 men tested and subsequently circumcised as of December 2013 although this falls short of the targeted 80% circumcision coverage (1,912,595) among men aged 15–49 years [44]. We therefore anticipate that HIV testing will increase significantly among men as the nation strives to reach its 80% coverage targets.

Conducting HIV testing campaigns or using mobile VCT teams [45] can also be a feasible and acceptable strategy that can be targeted at increasing testing among men, the younger age groups and the lower-educated and lower income-level populations although cost implications will play a role in assessing their feasibility. Also given that HIV-related behaviour change communication through mass media interventions has been shown to positively impact on knowledge of HIV transmission and reduction in high-risk sexual behaviour [46], these strategies can help improve HIV testing when disseminated with HTC communication. Finally, the fairly high proportion of individuals with undiagnosed HIV highlights the need for HIV-testing among first-timers and also re-testing among those who may have previously tested HIV-negative so that these individuals can access HIV treatment and care services. Whilst blanket approaches to increase HIV testing may have been adopted and scaled up, these approaches are clearly missing those HIV-infected and therefore it is necessary to develop HIV testing strategies targeted at HIV high-risk group populations and so called HIV “hot-spot” regions. Operational research to determine the magnitude of leakages in the cascade from HIV testing to entry into HIV treatment and care services and subsequent ART initiation among those HIV-infected are essential in providing useful information to policy makers in order to address this problem.

### Disclaimer

The views expressed are those of the authors and do not necessarily reflect the views of USAID, CDC or the United States Government. ICF international, Calverton, Maryland, USA, provided limited technical assistance for the analysis and preparation of the paper.
